# Psychosocial and Behavioral Correlates of HIV Serostatus Among Men Who Have Sex with Men in Portugal: A Cross-Sectional Study

**DOI:** 10.3390/healthcare14142164

**Published:** 2026-07-17

**Authors:** Felipe Alckmin-Carvalho, Iara Teixeira, Fernanda Lopes Guedes, Emerson Do Bú, Henrique Pereira, Martim Santos, Guilherme Wendt

**Affiliations:** 1 Department of Psychology and Education, Faculty of Social and Human Sciences, University of Beira Interior, 6200-209 Covilhã, Portugal; fer.guedes@ubi.pt (F.L.G.); hpereira@ubi.pt (H.P.); martimsantos@email.com (M.S.); 2 Research Center in Sports Sciences, Health Sciences and Human Development (CIDESD), 5001-801 Vila Real, Portugal; 3 Psychology Research Center, School of Psychology, University of Minho, 4710-057 Braga, Portugal; iarateixeiraint@gmail.com; 4 Institute of Social Sciences, University of Lisbon, 1600-189 Lisbon, Portugal; emerson.bu@edu.ulisboa.pt; 5 RISE-Health, Department of Psychology and Education, Faculty of Social and Human Sciences, University of Beira Interior, 6200-209 Covilhã, Portugal; 6 Department of Medical Sciences, Western Paraná State University, Francisco Beltrão 85605-010, PR, Brazil; guilherme.wendt@unioeste.br

**Keywords:** HIV serostatus, men who have sex with men (MSM), minority stress, compulsive sexual behavior, internalized homonegativity, substance use, chemsex, emotion regulation, psychological distress, syndemics, sexual health, Portugal

## Abstract

**Background/Objectives**: Men who have sex with men (MSM) carry a disproportionate share of the HIV burden in Portugal, yet the psychosocial characteristics of MSM living with HIV remain insufficiently described. This cross-sectional study examined how compulsive sexual behavior, internalized homonegativity, psychological distress, emotion regulation difficulties, and substance use relate to HIV serostatus, independent of sociodemographic characteristics. **Methods**: A convenience sample of 480 cisgender MSM (66 living with HIV) completed validated self-report measures online. Because the measures departed from normality, group differences were tested with Mann–Whitney U tests, and associations with serostatus were estimated with a parsimonious binary logistic regression, re-estimated with Firth penalization and evaluated with discrimination diagnostics. **Results**: Participants living with HIV showed small but significant differences on four indicators: higher compulsive sexual behavior and internalized homonegativity and lower scores on one emotion regulation subscale; depression, anxiety, and stress did not differ. In the adjusted model, compulsive sexual behavior, internalized homonegativity, and problematic substance use were associated with higher odds and the emotion regulation subscale with lower odds of living with HIV. In sensitivity analyses, the substance use association depended on how the construct was defined and was specific to chemsex with core drugs. Model discrimination was limited. **Conclusions**: HIV serostatus was associated with a small set of psychosocial and behavioral correlates rather than sociodemographic characteristics. These group-level associations describe correlates of serostatus, not a clinical profile of men living with HIV; they cannot establish direction and generate hypotheses, for longitudinal and structurally informed research, about stigma and social context.

## 1. Introduction

Human immunodeficiency virus (HIV) is a chronic infection that, without timely diagnosis and treatment, progressively weakens the immune system and can advance to acquired immunodeficiency syndrome (AIDS) [1]. With effective antiretroviral treatment, however, HIV has become a manageable chronic condition, and the central challenges have shifted toward early diagnosis, sustained engagement in care, and the social and structural conditions that shape them [1].

Diagnosis and treatment have improved substantially worldwide [1], yet the epidemic remains a major public health problem. The Joint United Nations Program on HIV/AIDS (UNAIDS) estimated that, in 2024, approximately 40.8 million people were living with HIV, including 1.3 million newly infected individuals. While 87% of people living with HIV knew their status and 77% were on treatment, about 5.3 million individuals remained undiagnosed, limiting progress toward epidemic control [1]. Since the onset of the epidemic, over 91 million people have acquired HIV, and roughly 44 million have died from AIDS-related causes [1]. Although mortality has declined markedly since the mid-2000s peak, the 630,000 AIDS-related deaths recorded in 2024 remain considerably above international targets [1,2]. The persistence of late diagnosis, with an estimated 1.8 million people currently living with advanced HIV disease, further reflects the structural and social barriers that delay early access to care [2].

Portugal has seen comparable progress, although important gaps remain. National surveillance data indicate that 924 new HIV cases were reported in 2023, with a 36% decrease in new HIV diagnoses and a 66% decline in new AIDS cases since 2014 [3]. Despite these improvements, the epidemic remains highly concentrated among men, particularly men who have sex with men (MSM), who represented 61.6% of new diagnoses among men in 2023 [3]. Persistent late diagnosis, evidenced by the 128 new AIDS cases and 111 AIDS-related deaths reported that year, points to gaps in testing uptake, healthcare access, and social acceptance that may shape health-seeking behaviors.

The World Health Organization (WHO) and UNAIDS have established the goal of ending AIDS as a public health threat by 2030. Achieving the 95–95–95 targets (i.e., diagnosis, treatment, and viral suppression) is essential for meeting this objective [4,5]. However, these goals cannot be met without addressing the disproportionate burden shouldered by key populations, particularly MSM, sex workers, people who use drugs, incarcerated individuals, and transgender people. These groups face HIV prevalence rates several times higher than the general population, shaped largely by social and structural determinants, including discrimination, criminalization, stigma, and restricted access to prevention and treatment services [1,6]. Understanding the psychosocial, behavioral, and contextual factors associated with HIV serostatus in each key population is therefore needed to design targeted interventions and inform equitable public health policy.

In Portugal, epidemiological trends indicate particular vulnerabilities among MSM, with national surveillance systems and strategic documents increasingly acknowledging the relevance of stigma and discrimination. Nevertheless, both public health surveillance and national research have remained focused primarily on epidemiological monitoring, HIV testing, treatment engagement, and biomedical prevention indicators [3,5,6]. A recent scoping review of HIV prevention research in Portugal highlighted that existing studies predominantly focus on isolated prevention outcomes, such as HIV testing, pre-exposure and post-exposure prophylaxis (PrEP/PEP) use, and transmission-related indicators, often within specific key populations (e.g., immigrants, sex workers) and without integrating broader psychosocial dimensions [7]. Accordingly, studies conducted in Portugal involving MSM have mainly addressed sexual behavior patterns and sexually transmitted infection (STI) risk [8], as well as molecular epidemiology and transmission cluster dynamics [9]. In contrast, psychosocial dimensions have received comparatively limited attention and have been explored mostly through isolated qualitative studies focused on stigma and lived experiences related to HIV [10]. Context-specific research integrating psychosocial, behavioral, and minority stress-related factors associated with HIV serostatus among MSM in Portugal therefore remains scarce.

Co-occurring psychosocial burdens also shape HIV-related outcomes among MSM, and they are often conceptualized within syndemic frameworks. The Syndemic Theory proposes that certain health conditions, such as HIV/AIDS, do not occur in isolation but cluster together and interact in contexts of stark social inequality, and this mutual interaction reinforces their negative effects on people’s well-being. Rather than considering only comorbidities, the syndemic perspective emphasizes that physical illnesses, mental health conditions, and psychosocial issues, such as violence, substance misuse, stigma, and discrimination, are exacerbated in environments characterized by poverty, racism, sexism, homophobia, and other forms of structural oppression. Evidence from diverse contexts supports this framework: sexualized drug use, including chemsex, has been repeatedly linked to a higher likelihood of condomless anal sex, multiple sexual partners, STI acquisition, and distinct sexual networking patterns associated with higher HIV exposure [11,12,13], and chemsex engagement also shapes the uptake of biomedical prevention such as pre-exposure prophylaxis [14]. Similarly, compulsive or dysregulated sexual behavior has been associated with elevated STI and HIV incidence and with broader syndemic clusters involving problematic substance use, violence victimization, and psychological distress [15,16].

Minority Stress Theory [17] offers a complementary framework for understanding the psychosocial factors that contribute to HIV vulnerability in sexual minority populations. According to this model, stigma-related processes, including discrimination, the expectation of rejection, and the internalization of homonegativity, can compromise mental health, encourage maladaptive coping, and reduce engagement with prevention and care services. Internalized homonegativity has been associated with lower uptake of HIV testing and with HIV risk behaviors [18], and the broader empirical literature on this construct has been systematically mapped [19]; ongoing work is examining how internalized homonegativity intersects with other internalized stigmas, such as racism, among multiply marginalized men [20]. Among younger MSM, cumulative psychosocial burdens, including mental distress, substance use, discrimination, and sexual victimization, are linked to riskier sexual behaviors, including patterns of partner selection within digital environments [21,22]. Across these studies, the psychosocial and behavioral factors associated with HIV serostatus among MSM operate at several levels; it is nonetheless important to distinguish factors associated with HIV acquisition risk from correlates of established HIV serostatus, particularly in cross-sectional designs.

Less is known about the way these psychosocial factors are distributed across national and cultural contexts, particularly among key populations such as MSM. This study therefore examined whether MSM living with HIV in Portugal differ from HIV-negative MSM on psychosocial, behavioral, and sociodemographic variables. It had three aims: (1) to compare levels of compulsive sexual behavior, internalized homonegativity, psychological distress, emotion regulation difficulties, and substance use between MSM living with and without HIV; (2) to evaluate associations among these psychosocial and behavioral variables; and (3) to identify which psychosocial and behavioral factors are most strongly associated with HIV serostatus in a cross-sectional sample, after controlling for sociodemographic characteristics. Consistent with minority stress and syndemic perspectives, we expected the psychosocial burdens (compulsive sexual behavior, internalized homonegativity, psychological distress, and emotion regulation difficulties) to show positive intercorrelations, and we expected the same burdens to be associated with HIV serostatus; we treat these as directional expectations to be examined rather than as formal hypotheses tested with confirmatory procedures. Throughout the paper, the psychosocial and behavioral measures are treated as correlates shaped by stigma and social context rather than as deficits attributable to men living with HIV. All findings reported here are cross-sectional associations; they are intended to motivate future longitudinal and structurally informed research rather than to support individual-level or causal inferences.

## 2. Materials and Methods

This cross-sectional study compared cisgender Portuguese MSM by HIV serostatus on a set of psychosocial, behavioral, and sociodemographic characteristics.

### 2.1. Participants

Recruitment produced a non-probabilistic convenience sample. Eligibility was limited to cisgenderMSM, who are at least 18 years old, who have resided in Portugal for at least one year, and with the internet access and literacy needed to complete an online questionnaire; no probability sampling, quotas, or weighting were applied. Of the 562 records collected, 81 were excluded because the respondent was not a cisgender man or did not report a known recent HIV result, since serostatus could not otherwise be placed within a comparable timeframe, and one further respondent who reported being under 18 was excluded. No formal sensitivity analysis compared excluded and retained cases. The analytic sample comprised 480 cisgender MSM, of whom 66 (13.8%) reported living with HIV and 414 (86.3%) reported a negative result.

### 2.2. Instruments

***Living with HIV*** was self-reported through two questions from the sociodemographic questionnaire: whether the respondent had tested for HIV in the past year (yes or no) and the result of the most recent test (positive or negative). Respondents who reported a test in the past year and a positive result were classified as living with HIV.

***Hypersexual Behavior Inventory.*** This 19-item self-report scale [23], validated for Portuguese speakers by Alckmin-Carvalho et al. [24], indexes perceived difficulty regulating sexual behavior across three subscales, and the Portuguese version is bifactorial. The first factor, control and consequences, captures how far a person feels unable to rein in their sexual behavior and perceives related harm to physical and mental health and to quality of life. The second factor, coping, reflects using sex to manage negative emotions or stressful events despite those perceived consequences. Items are rated from 1 (never) to 5 (very often), and total scores range from 19 to 95; scores above 53 fall in the clinical range and warrant further assessment. Internal consistency was high in the original (α = 0.91) and in the Portuguese version (α = 0.94).

***Internalized Homonegativity Scale.*** This scale [25] measures internalized negative attitudes toward one’s own homosexuality across two dimensions, perceived social oppression and internalized stigma. Items are affirmative and rated from 1 (strongly disagree) to 4 (strongly agree), and higher scores reflect greater internalized homonegativity, with no clinical cutoff. The present study used the 19-item internalized-stigma subscale (α = 0.82), with scores ranging from 19 to 76 [26].

***Depression, Anxiety, and Stress Scales (DASS-21, reduced version).*** This 21-item version assigns seven items to each of three subscales, depression, anxiety, and stress, with each item scored from 0 (“does not apply to me at all”) to 3 (“applies to me most of the time”); subscale sums are then doubled. The resulting scores place respondents into severity bands for anxiety (normal 0–7, mild 8–9, moderate 10–14, severe 15–19, extremely severe ≥ 20), depression (normal 0–9, mild 10–13, moderate 14–20, severe 21–27, extremely severe ≥ 28), and stress (normal 0–14, mild 15–18, moderate 19–25, severe 26–33, extremely severe ≥ 34). Reliabilities were adequate in the original (α = 0.91 for depression, 0.84 for anxiety, 0.90 for stress) and in the Portuguese version (α = 0.85, 0.74, and 0.81) [27,28].

***Difficulties in Emotion Regulation Scale, Short Form (DERS-SF).*** This self-report instrument [29] assesses difficulties across six domains: nonacceptance, goals, impulses, awareness, strategies, and clarity. The 18-item Portuguese version [30] reproduced this multidimensional structure and showed internal consistency from α = 0.70 to 0.91 and convergent validity with emotional symptoms. Each domain holds three items; subscale scores sum their items, and the total score, the sum of all items, reflects overall emotion regulation difficulty, with higher scores indicating greater dysregulation.

***Sociodemographic and Clinical Questionnaire.*** We built this questionnaire for the present study. It covered sociodemographic characteristics (age, gender, sexual orientation, marital status, education, occupation, employment status, monthly income, household size, city size, economic vulnerability, and religious or spiritual practice) and clinical characteristics (age at diagnosis, antiretroviral therapy, most recent viral load, problematic or harmful use of alcohol, cannabis, cocaine, or other illicit substances, and the use of substances to intensify, prolong, or disinhibit sexual activity).

### 2.3. Procedures

This study was advertised on Facebook and Instagram over three months, from August to October 2025, with paid promotion used to widen reach. The online assessment protocol, built by the first author for this study, checked the inclusion criteria on its opening page, and only eligible respondents continued to the measures. Completing the protocol took about 30 min, and participants received no financial compensation.

### 2.4. Ethical Aspects

The Ethics Committee for Research with Human Subjects of the Graduate Program in Clinical and Health Psychology at the Faculty of Social and Human Sciences, University of Beira Interior, approved the study on 15 October 2024 (approval number CE-UBI-Pj-2024-086-ID2760). The informed consent form appeared on the opening page of the online protocol, and only participants who gave electronic consent continued to the questionnaires.

Participation was voluntary and anonymous: the protocol recorded no names or contact details and collected no direct identifiers, so individual responses could not be traced back to participants.

### 2.5. Data Analysis

Analyses were conducted in R (version 4.5), and the analysis scripts, codebook, and session information are openly available in the study’s Open Science Framework repository. The analytic sample comprised 480 cisgender MSM with a known HIV serostatus; one participant who reported being younger than 18 was excluded. Categorical variables were summarized as frequencies and percentages and continuous variables as means and standard deviations.

Group differences by serostatus were tested with chi-square or Fisher’s exact tests for categorical variables. For the continuous psychosocial measures, the assumption of homogeneity of variance was checked with Levene’s test and distributional shape with the Shapiro–Wilk test. Because most measures departed from normality and some showed unequal variances, group differences were evaluated with the non-parametric Mann–Whitney U test. Cohen’s d [31] is reported alongside it as a descriptive effect size measure. Effect sizes for categorical associations were the phi coefficient for 2 × 2 tables. Substance use was operationalized in four complementary ways, all based on a self-report: an indicator of problematic substance use and three progressively broader sexualized use indicators, labeled chemsex (any substance) for the use of any substance to enable or enhance sex in the past year, chemsex with core drugs for use restricted to the classic chemsex drugs (methamphetamine, mephedrone and related cathinones, and GHB/GBL), and chemsex with core or additional drugs, which adds ketamine, cocaine, and MDMA to that list.

Associations with serostatus were estimated with a single parsimonious binary logistic regression that included five theory-driven correlates (compulsive sexual behavior, internalized homonegativity, problematic substance use, the Strategies subscale of the DERS-SF, and age), keeping the number of events per estimated parameter within recommended limits [32]; logistic regression does not assume normally distributed predictors. Because the number of events was modest, the model was re-estimated with Firth’s penalized likelihood to guard against small sample and separation bias, and multicollinearity was assessed with variance inflation factors. Discrimination and calibration were summarized with the area under the ROC curve, with sensitivity and specificity at the conventional 0.50 threshold and at the Youden-optimal threshold, with balanced accuracy, and with the Hosmer–Lemeshow test; overall classification accuracy was read against the marked imbalance in serostatus rather than on its own. The sensitivity of the substance use association to its definition was examined by substituting each alternative indicator into the adjusted model. All tests were two-sided, with α = 0.05.

## 3. Results

### 3.1. Sample Characteristics and Group Comparisons

The analytic sample comprised 480 cisgenderMSM, 66 of whom were living with HIV and 414 of whom were HIV-negative. The two groups were comparable on most sociodemographic characteristics, including age, education, employment, income, area of residence, economic vulnerability, and religious affiliation and practice (Table 1). For a broad indicator of chemsex (any substance), the groups did not differ (χ^2^(1) = 1.16, *p* = 0.281). Significant differences were confined to more specific indicators: problematic substance use was more frequent among participants living with HIV (18.2% vs. 7.8%, χ^2^(1) = 7.34, *p* = 0.007), as was chemsex with core drugs (12.9% vs. 4.2%, Fisher’s exact *p* = 0.010) and chemsex with core or additional drugs (14.5% vs. 6.2%, Fisher’s exact *p* = 0.033) (Table 1).

On the continuous psychosocial measures, the Mann–Whitney U test identified significant group differences on four indicators, all small in magnitude. Participants living with HIV scored higher on compulsive sexual behavior on both its total score (*p* = 0.024) and its control/consequences facet (*p* = 0.041) and higher on internalized homonegativity (*p* = 0.014); they scored lower on the Strategies subscale of the Difficulties in Emotion Regulation Scale (*p* = 0.025), indicating fewer reported difficulties on that subscale. Depression, anxiety, and stress did not differ between groups, nor did the coping facet of compulsive sexual behavior or the remaining emotion regulation subscales (Table 2).

### 3.2. Correlations Among Psychosocial Measures

**Figure 1:**
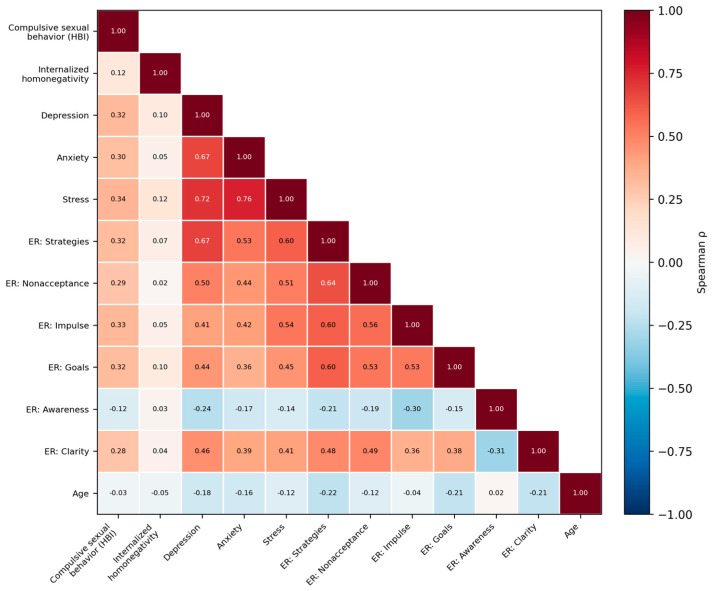
Spearman correlations among psychosocial measures. Note. ER = emotion regulation difficulties (DERS-SF subscales); HBI = Hypersexual Behavior Inventory. Coefficients are Spearman rank-order correlations computed on available cases.

Spearman correlations among the psychosocial measures are displayed in Figure 1. Broadly consistent with expectations, many affective and self-regulatory burdens were positively intercorrelated: the depression, anxiety, and stress subscales were strongly related (ρ = 0.67 to 0.76; e.g., anxiety with stress, ρ = 0.76, *p* < 0.001); the emotion regulation subscales were mostly positive but heterogeneous, with negative coefficients involving awareness (ρ = −0.31 to 0.64); and compulsive sexual behavior correlated with both psychological distress (ρ = 0.30 to 0.34; e.g., with stress, ρ = 0.34, *p* < 0.001) and emotion regulation difficulties (ρ = 0.28 to 0.32; e.g., with the Strategies subscale, ρ = 0.32, *p* < 0.001). Contrary to expectation, internalized homonegativity was largely independent of the other measures, with weak and mostly non-significant correlations (with compulsive sexual behavior, ρ = 0.12, *p* = 0.010; with stress, ρ = 0.12, *p* = 0.008; with the Strategies subscale, ρ = 0.07, *p* = 0.145; with anxiety, ρ = 0.05, *p* = 0.259). Age correlated negatively and weakly with distress and emotion regulation difficulties (e.g., with the Strategies subscale, ρ = −0.22, *p* < 0.001). All variance inflation factors in the regression were below 1.3, indicating no multicollinearity. The expected positive intercorrelations among the psychosocial burdens (aim 2) were thus confirmed for distress, most emotion regulation domains, and compulsive sexual behavior, but not for internalized homonegativity.

### 3.3. Correlates of HIV Serostatus

Associations with HIV serostatus were estimated with a parsimonious binary logistic regression that included five theory-driven correlates: compulsive sexual behavior, internalized homonegativity, problematic substance use, the DERS Strategies subscale, and age. The 65 events yielded approximately 13 events per estimated parameter, within the range recommended for stable logistic estimation. Given the modest number of events, the model was re-estimated with Firth’s penalized likelihood; the penalized and maximum likelihood estimates were materially identical, which indicates that the coefficients are not artifacts of separation or small sample bias (Table 3).

Higher compulsive sexual behavior and higher internalized homonegativity were each associated with higher odds of living with HIV, and both associations persisted after mutual adjustment and adjustment for age (Table 3). Problematic substance use was likewise associated with higher odds of living with HIV; this estimate rests on a small number of affirmative responses and is therefore best read as exploratory (Table 3). Higher scores on the DERS Strategies subscale were associated with lower odds of living with HIV, and age was not associated with serostatus.

The substance use association was sensitive to how chemsex was defined (Table 4). The broad indicator, chemsex (any substance), was not associated with serostatus, whereas the indicator restricted to chemsex with core drugs (methamphetamine, mephedrone and related cathinones, and GHB/GBL) was associated with higher odds of living with HIV. Chemsex with core or additional drugs, which additionally counts ketamine, cocaine, and MDMA, was not associated at the 0.05 level. A single broad item that lumps all sexualized substance use together can therefore mask an association that is specific to chemsex with core drugs.

Overall model fit was modest and discrimination was limited (Table 3 and Table 5). At the conventional probability cut point of 0.50, classification accuracy was high, but this value simply tracked the large majority of HIV-negative participants: an uninformative rule that classifies everyone as HIV-negative reaches the same accuracy, and the fitted model identified almost none of the participants who were living with HIV (Table 5). At a cut point selected to balance sensitivity and specificity, the two indices were more even but remained modest (Table 5). Calibration was adequate. These results indicate that the model characterizes group-level associations and does not provide a basis for identifying which individuals are living with HIV.

## 4. Discussion

This study examined the psychosocial and behavioral characteristics associated with HIV serostatus among cisgender MSM in Portugal. Participants living with HIV differed from their HIV-negative peers on a small number of measures, the associations were modest in size, and they did not extend to sociodemographic characteristics or to general psychological distress. Because the design is cross-sectional, these associations cannot be read as causes or consequences of serostatus; several are at least as consistent with the experience of living with HIV in a stigmatizing context as with any factor that preceded infection. We therefore frame the findings as correlates to be understood at the group level, not as a clinical profile that characterizes individuals living with HIV.

### 4.1. Group Differences in Psychosocial and Behavioral Characteristics

Participants living with HIV reported higher compulsive sexual behavior than HIV-negative participants, with a small effect. The instrument captures self-perceived difficulty regulating sexual thoughts and behavior rather than the frequency of any specific sexual practice, so the difference describes how participants appraised their own sexual self-regulation and does not index sexual risk behavior. Internalized homonegativity was likewise modestly higher among participants living with HIV. This pattern is consistent with minority stress accounts, in which the stigma attached to both sexual minority status and HIV is internalized over time [17] through identifiable psychological processes [33], and elevated internalized homonegativity among people living with HIV has been reported in other settings [34,35].

General psychological distress did not differ between groups. Among the emotion regulation domains, only the Strategies subscale distinguished the groups, and it did so in the direction of fewer reported difficulties among participants living with HIV. We interpret this isolated and counterintuitive difference cautiously: it may reflect adaptation or self-management developed through living with a chronic condition and engaging with care, but with a single subscale reaching significance and a small effect, it could also be a chance finding and requires replication.

### 4.2. Interrelations Among the Psychosocial Measures

The expectation that the psychosocial burdens would be positively intercorrelated was only partly borne out. Distress and most emotion regulation domains cohered as anticipated, and compulsive sexual behavior was modestly tied to both, consistent with accounts that link dysregulated sexual behavior to broader affective and self-regulatory difficulties [36,37,38]. Internalized homonegativity, however, correlated only weakly with the other measures, contrary to a reading in which internalized stigma and general distress move together. One interpretation is that internalized homonegativity, as a distal minority stress process, is conceptually and empirically separable from the proximal affective states captured by the distress and emotion regulation scales [17,33]; it need not co-vary strongly with them to be associated with serostatus, as the regression indicated. This separation cautions against treating internalized homonegativity as interchangeable with psychological distress in either measurement or intervention, and it is a pattern that longitudinal work could test directly.

### 4.3. Correlates of HIV Serostatus and Their Interpretation

When the correlates were considered together, higher compulsive sexual behavior, higher internalized homonegativity, and problematic substance use were each associated with higher odds of living with HIV, and the Strategies subscale was associated with lower odds. The substance use association was sensitive to how the construct was defined: a broad indicator, chemsex (any substance), was not associated with serostatus, whereas an indicator restricted to chemsex with core drugs was. This pattern fits a literature in which a specific chemsex repertoire, rather than sexualized substance use in general, is associated with HIV [12,39], and it rests here on a small number of affirmative responses, so we treat it as exploratory.

The model characterized group-level associations but did not discriminate well between individuals. Overall classification accuracy was high only because most participants were HIV-negative; at a balanced threshold the model identified people living with HIV only modestly better than chance. These correlates therefore do not constitute a screening signature, and we do not read them as identifying individuals who are living with HIV. One plausible structural interpretation is that experiences such as internalized stigma and patterns of substance use are shaped by the social conditions that also shape HIV exposure, undiagnosed infection, and access to prevention and care [40,41,42]. Because these structural conditions, including enacted and anticipated stigma, were not measured directly in this study, this interpretation is offered as a hypothesis rather than a demonstrated pathway. Accordingly, the directions these findings suggest, such as reducing stigma, supporting affirmative services and integrating chemsex-aware care into sexual health services [43], are best framed as hypotheses and lines of research to be evaluated in studies designed to measure structural conditions directly, rather than as conclusions established by the present data or as individual psychological screening, which the data do not support. Read together, these correlates describe statistical associations with serostatus in a single sample; they do not portray men living with HIV as defined by compulsive sexuality, internalized homonegativity, or substance use, and they should not be taken to locate the drivers of HIV within individuals rather than in the structural conditions that shape exposure and adaptation.

### 4.4. Limitations

Several limitations qualify these conclusions. The cross-sectional design precludes any inference about temporal order or causation. Data were self-reported and collected through an online convenience sample, which limits representativeness and may introduce recall and social desirability biases; HIV serostatus itself was self-reported. The substance use and chemsex indicators were derived from a limited set of items and do not capture frequency, quantity, or the sexual context of use in detail. The analysis did not include proximal HIV-acquisition variables such as condomless anal sex, pre-exposure prophylaxis use, or viral suppression, which lie outside the present aims but are central to risk. Finally, the sample comprised cisgender men, so the findings do not extend to transgender or non-binary people, and the number of participants living with HIV was modest, which constrained model complexity and statistical power. The discrimination and classification results reported for the adjusted model (Table 5) were obtained from the same sample used to estimate the model, so they describe its apparent, in-sample performance and probably overstate how well it would separate serostatus groups in new respondents. Because the model was neither refitted in an independent sample nor internally validated through resampling, its true discriminatory capacity cannot be established here and should be examined in future work using separate or cross-validated data. A further limitation is conceptual. Stigma, minority stress, and the structural conditions we drew on to interpret the pattern of correlates were not measured directly, and neither were the proximal exposures that shape HIV acquisition. For that reason, the readings we offer in terms of internalized and structural stigma and any implication for stigma reduction, affirmative care, or chemsex-sensitive services are best treated as hypotheses and directions for research rather than as findings that follow from the present data.

## 5. Conclusions

Among cisgender MSM in Portugal, living with HIV was associated with modestly higher compulsive sexual behavior and internalized homonegativity, with problematic substance use, and with one emotion regulation subscale, but not with general psychological distress or with sociodemographic characteristics. In sensitivity analyses, the substance use association was specific to chemsex with core drugs. These associations were small and were estimated from a cross-sectional, convenience sample with self-reported serostatus and a modest number of participants living with HIV; they did not distinguish individuals well enough to serve as a screening profile, and these design features limit the weight the findings can bear. They are best understood as group-level correlates embedded in the social and structural context of living with HIV. Rather than supporting specific clinical recommendations, they generate hypotheses about stigma reduction, affirmative care, and chemsex-aware sexual health services for longitudinal and structurally informed research designed to measure these conditions directly and to clarify direction and mechanisms.

## Figures and Tables

**Table 1:** Sample characteristics by HIV serostatus (*N* = 480).

Characteristic	Living with HIV	HIV-Negative	Test	Effect Size
	(*n* = 66)	(*n* = 414)		
Age (years), *M (SD)*	43.68 (11.22)	40.74 (12.63)	t (92.1) = 1.93, *p* = 0.057	0.24
Education			χ^2^ (1) = 0.47, *p* = 0.493	0.03
Up to higher education (incomplete)	11 (16.7)	84 (20.3)		
Higher education	55 (83.3)	330 (79.7)		
Employment			Fisher *p* = 0.393	0.04
Employed	56 (91.8)	333 (94.3)		
Unemployed	5 (8.2)	20 (5.7)		
Monthly income			χ^2^ (2) = 1.11, *p* = 0.575	0.05
≤1200 EUR	30 (46.2)	166 (40.1)		
1201–2000 EUR	20 (30.8)	130 (31.4)		
>2000 EUR	15 (23.1)	118 (28.5)		
Area of residence			χ^2^ (2) = 5.44, *p* = 0.066	0.11
Village/small town	7 (10.6)	93 (22.5)		
Medium city	14 (21.2)	91 (22.0)		
Large city	45 (68.2)	230 (55.6)		
Economic vulnerability (yes)	7 (10.9)	33 (8.2)	χ^2^ (1) = 0.53, *p* = 0.465	0.03
Religion			χ^2^ (2) = 1.00, *p* = 0.606	0.05
Catholic	23 (34.8)	127 (30.7)		
Other	11 (16.7)	59 (14.3)		
None	32 (48.5)	228 (55.1)		
Religious practice			Fisher *p* = 0.526	0.06
Frequent	3 (4.6)	30 (7.2)		
Occasional	12 (18.5)	56 (13.5)		
None	50 (76.9)	328 (79.2)		
Problematic substance use (yes)	12 (18.2)	32 (7.8)	χ^2^ (1) = 7.34, *p* = 0.007	0.12
Chemsex, any substance (yes)	16 (24.2)	77 (18.6)	χ^2^ (1) = 1.16, *p* = 0.281	0.05
Chemsex with core drugs (yes)	8 (12.9)	16 (4.2)	Fisher *p* = 0.010	0.13
Chemsex with core or additional drugs (yes)	9 (14.5)	24 (6.2)	Fisher *p* = 0.033	0.11

Note. Values are *n* (%) unless otherwise indicated; percentages are column percentages within available cases. Chemsex variables have missing data because the substance type was not always specified; chemsex with core drugs covers methamphetamine, mephedrone or related cathinones, and GHB/GBL, and chemsex with core or additional drugs additionally includes ketamine, cocaine, and MDMA. Effect size is Cohen’s d for age, φ for 2 × 2 tables, and Cramér’s V otherwise.

**Table 2:** Group comparisons on psychosocial measures by HIV serostatus.

Measure	Living with HIV	HIV-Negative	Mann–Whitney	d [95% CI]
	*M (SD)*	*M (SD)*	*p*	
Compulsive sexual behavior (HBI total)	45.24 (18.64)	39.64 (15.98)	0.024	0.34 [0.08, 0.60]
HBI: control/consequences	26.38 (12.42)	22.82 (9.93)	0.041	0.35 [0.08, 0.61]
HBI: coping	18.86 (8.36)	16.82 (7.50)	0.068	0.27 [0.01, 0.53]
Internalized homonegativity	61.48 (5.01)	59.86 (5.11)	0.014	0.32 [0.06, 0.58]
Depression (DASS-21)	6.73 (5.41)	7.56 (5.95)	0.350	−0.14 [−0.40, 0.12]
Anxiety (DASS-21)	5.15 (5.29)	5.45 (5.14)	0.470	−0.06 [−0.32, 0.20]
Stress (DASS-21)	8.74 (4.94)	8.73 (5.34)	0.799	0.00 [−0.26, 0.26]
ER: strategies	7.15 (2.87)	8.10 (3.12)	0.025	−0.31 [−0.57, −0.05]
ER: nonacceptance	7.92 (3.50)	8.51 (3.51)	0.199	−0.17 [−0.43, 0.09]
ER: Impulse	6.98 (3.10)	6.88 (3.01)	0.801	0.04 [−0.22, 0.30]
ER: Goals	9.77 (3.24)	10.39 (3.18)	0.138	−0.19 [−0.45, 0.07]
ER: awareness	11.68 (2.18)	11.72 (2.22)	0.851	−0.02 [−0.28, 0.24]
ER: Clarity	6.33 (2.92)	6.71 (2.91)	0.297	−0.13 [−0.39, 0.13]

Note. Group differences were tested with the Mann–Whitney U test; the two-sided *p* value from the Wilcoxon rank-sum test is reported. Levene’s and Shapiro–Wilk tests indicated unequal variances and departures from normality for several measures, so the non-parametric test is reported as primary. d = Cohen’s d, reported as a descriptive effect size measure (positive values indicate higher scores among participants living with HIV). HBI = Hypersexual Behavior Inventory; DASS-21 = Depression, Anxiety and Stress Scales; ER = emotion regulation difficulties (DERS-SF).

**Table 3:** Unadjusted and adjusted associations with HIV serostatus (binary logistic regression).

Predictor	Unadjusted OR [95% CI]	Adjusted OR [95% CI]	*p* (Adjusted)
Compulsive sexual behavior (HBI total)	1.02 [1.00, 1.03]	1.03 [1.01, 1.05]	0.001
Internalized homonegativity	1.07 [1.01, 1.12]	1.07 [1.01, 1.13]	0.020
Problematic substance use	2.63 [1.28, 5.42]	2.89 [1.32, 6.31]	0.008
ER: strategies	0.90 [0.82, 0.99]	0.84 [0.76, 0.94]	0.002
Age	1.02 [1.00, 1.04]	1.02 [1.00, 1.04]	0.101

Note. Adjusted estimates are from a single multivariable model (*n* = 467, 65 events); unadjusted estimates are from separate single-predictor models. Firth penalized likelihood estimates were materially identical to the maximum likelihood estimates (e.g., problematic substance use adjusted OR = 2.88, 95% CI [1.31, 6.12]). Model fit: Nagelkerke R^2^ = 0.12; area under the ROC curve = 0.69, 95% CI [0.62, 0.76]; Hosmer–Lemeshow χ^2^ (8) = 11.31, *p* = 0.185. All variance inflation factors were below 1.3. OR = odds ratio; CI = confidence interval; HBI = Hypersexual Behavior Inventory; ER = emotion regulation difficulties (DERS-SF).

**Table 4:** Sensitivity of the substance use association to the way chemsex is defined.

Substance Indicator	*n*	Events	Adjusted OR	95% CI	*p*
Problematic substance use	467	65	2.89	[1.32, 6.31]	0.008
Chemsex (any substance)	470	65	1.09	[0.56, 2.13]	0.801
Chemsex with core drugs (methamphetamine, cathinones, GHB/GBL)	438	61	2.87	[1.09, 7.52]	0.032
Chemsex with core or additional drugs (adding ketamine, cocaine, MDMA)	438	61	2.26	[0.95, 5.37]	0.065

Note. Each indicator replaces the problematic substance use term in the adjusted model in Table 3, holding the other predictors constant. The first row is the primary model term, shown for comparison. Firth penalized likelihood estimates were consistent. OR = odds ratio. Ninety-five percent confidence intervals are Wald intervals for the adjusted odds ratio; the interval for the primary problematic substance use term matches the value reported in Table 3.

**Table 5:** Discrimination and classification of the adjusted model.

Probability Threshold	Accuracy	Sensitivity	Specificity	Balanced Accuracy	Participants with HIV Correctly Classified
0.50 (conventional)	0.87	0.05	1.00	0.52	3 of 65
0.12 (Youden-optimal)	0.57	0.72	0.55	0.64	47 of 65

Note. Classification is based on the adjusted model in Table 3. The Youden-optimal cut point maximizes sensitivity plus specificity. A null rule that classifies every participant as HIV-negative reaches 0.86 accuracy. Area under the ROC curve = 0.69, 95% CI [0.62, 0.76]. These indices reflect apparent, in-sample performance and were not validated in independent or resampled data, so they likely represent an optimistic estimate of the model’s discriminatory capacity.

## Data Availability

The analysis scripts, codebook, and session information that support the findings are available in the Open Science Framework repository at https://doi.org/10.17605/OSF.IO/NJZK8. During peer review, these materials were available through an anonymized view-only link. The individual-level data are available from the corresponding author on reasonable request, given the privacy and ethical restrictions that apply to sensitive data from a sexual minority sample.

## Abbreviations

The following abbreviations are used in this manuscript:

HIV	Human immunodeficiency virus
MSM	Men who have sex with men
DASS-21	Depression, Anxiety and Stress Scales
DERS-SF	Difficulties in Emotion Regulation Scale—Short Form
HBI	Hypersexual Behavior Inventory
